# Digital preventive measures for arterial hypertension (DiPaH) – a mixed-methods study protocol for health services research

**DOI:** 10.3389/fcvm.2022.1089968

**Published:** 2023-01-10

**Authors:** Dunja Bruch, Felix Muehlensiepen, Susann May, Eileen Wengemuth, Olen Johannsen, Katrin Christiane Reber, Eva-Lotta Blankenstein, Gerrit Fleige, Martin Middeke, Johannes Albes, Martin Heinze, Marc Lehnen, Sebastian Spethmann

**Affiliations:** ^1^Department of Cardiovascular Surgery, Heart Center Brandenburg, Immanuel Klinikum Bernau, University Hospital Brandenburg Medical School (Theodor Fontane), Bernau bei Berlin, Germany; ^2^Faculty of Health Sciences, Brandenburg Medical School Theodor Fontane, Neuruppin, Germany; ^3^Center for Health Services Research, Brandenburg Medical School Theodor Fontane, Rüdersdorf bei Berlin, Germany; ^4^Hypertension Care UG, Ingolstadt, Germany; ^5^AOK Nordost – Die Gesundheitskasse, Strategische Versorgungsanalysen/GeWINO, Berlin, Germany; ^6^revFLect GmbH, Hanover, Germany; ^7^Brandenburg Medical School Theodor Fontane, Neuruppin, Germany; ^8^Hypertension Center Munich, Munich, Germany; ^9^Medical Department, Division of Cardiology and Angiology, Charité – Universitätsmedizin Berlin, Corporate Member of Freie Universität Berlin and Humboldt-Universität zu Berlin, Berlin, Germany

**Keywords:** hypertension, digital prevention measures, mixed-methods design, digital health literacy, rural regions

## Abstract

**Introduction:**

Digital health measures promise to further improve the quality of cardiovascular care but have not yet been widely implemented in routine care. The research project Digital preventive measures for arterial hypertension (DiPaH) will systematically identify structural and individual factors in different stakeholders that influence the use of digital preventive measures in patients with arterial hypertension in Germany. Special focus is given to remote and sparsely populated areas, the age-specific impact, as well as influence of digital health literacy.

**Methods and analysis:**

The DiPaH project is an exploratory cross-sectional study with a mixed-methods design, in which written surveys and interviews with patients and physicians will be conducted. In addition, secondary data from a health insurance company will be analyzed. In module 1, individuals from the database of the health insurance company with confirmed arterial hypertension will be interviewed (1,600 questionnaires, 30 interviews). Module 2 includes users of digital prevention offers and apps (400 questionnaires, 40 interviews) and in module 3, family physicians and cardiologists will be interviewed (400 questionnaires, 40 interviews). In a final module, the overall results will be analyzed and recommendations for interventions in clinical care will be derived.

**Discussion:**

The DiPaH project will contribute to a patient-oriented and demand-based improvement of arterial hypertension prevention services in health care. Challenges and barriers will be analyzed and the respective target groups identified based on their prevention needs and social characteristics to enable a patient-centered implementation of digital prevention of arterial hypertension and cardiovascular services in general, and finally to improve cardiovascular outcomes.

**Clinical trial registration:**

https://drks.de/search/de/trial/DRKS00029761, identifier DRKS00029761.

## Introduction

Despite effective guideline-based prevention and treatment options, cardiovascular diseases remain the leading cause of death worldwide (1). Arterial hypertension is one of the most important risk factors for cardiovascular diseases (2), affecting over one billion people worldwide (3). In addition to a genetic predisposition, the development of hypertension is strongly influenced by individual factors such as lifestyle (4), but also by local access to and quality of health care (5). In Germany, the prevalence of such risk factors varies greatly depending on the region. For example, obesity is more prevalent in the rural federal states (6). But it is precisely there that accessibility to medical care is limited due to a low density of doctors (7). As a result, hypertension-related diseases such as ischemic heart disease are most frequent in these states (8). On an individual level, there is also an association between hypertension and lower socioeconomic status and lower educational attainment (9). In summary, there are significant regional differences in Germany, with a higher incidence of arterial hypertension in socioeconomically disadvantaged areas (10).

**FIGURE 1:**
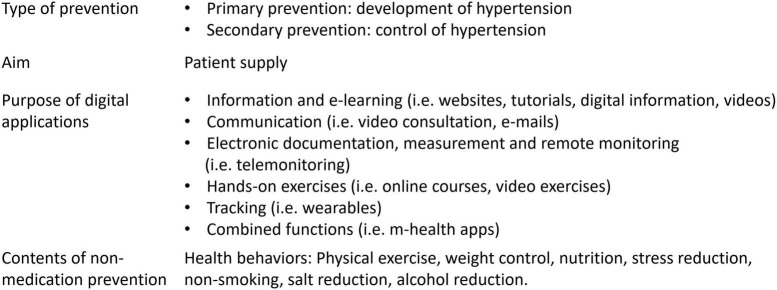
Characteristics of digital preventive measures for arterial hypertension.

In order to overcome these geographical disparities in health care and to strengthen individual health behavior even in regions with poor access to health care and aging population, digital prevention measures are an innovative and promising approach (11). These are digital applications such as remote monitoring (i.e., telemonitoring), e-learning, or m-health apps. The characteristics of digital preventive measures for arterial hypertension are shown in Figure 1. With telemonitoring, patient data is transmitted to the attending physicians (12). E-learning provides patients with information, e.g., on lifestyle modification through interactive and web-based educational material (12), and m-health apps are stand-alone software on a smartphone or tablet (13). Despite existing technical possibilities, digital health applications have so far only been insufficiently implemented (11), so that there is an increasing risk of a “digital divide” (14), whereby vulnerable groups with low digital health literacy are progressively excluded from innovative health offers. Current data (15) from Germany show low mobile Internet use among older people (only 36% of the population born up to 1945) and among people with lower educational attainment. Importantly, usage is lower in the more rural eastern German states than in the western states. Since people with lower incomes have less access to digital devices (15), one cause could be the lower gross average income in eastern Germany (16). However, people who are more limited in their mobility and have a higher risk of illness could particularly benefit from digital measures. Importantly, data on hypertension-specific digital applications are lacking so far.

Due to the importance of digital health interventions, the ESC e-Cardiology Working Group has published a position paper in 2019 addressing the challenges of digital health adoption in cardiovascular medicine (11). In addition to technical issues, there are also patient- and physician-related barriers [e.g., lack of information about available and qualified m-health apps (17), lack of digital skills (15)] as well as reimbursement and legal considerations to the introduction of digital health in cardiovascular medicine that need to be carefully analyzed and addressed prior to their successful deployment.

To overcome such barriers, the healthcare research project digital preventive measures for arterial hypertension (DiPaH) will therefore systematically identify individual, structural and application-related factors that influence the use of digital preventive measures in patients with arterial hypertension in Germany. Due to the regional differences already described, a particular focus of the study is on remote and sparsely populated areas, the age-specific effects as well as the influence of digital health literacy.

The study identifies not only general barriers to the use of digital prevention services, but also barriers specific to patient groups (e.g., with regard to motivation and health literacy). In order to take into account the complexity of prevention in statutory health insurance, the DiPaH study relates the experiences of patients and users of digital applications to the perspective of physicians. In this way, potentials, but also barriers that only emerge in the overall context of the health care system, can be identified and corresponding concrete recommendations for implementation can be derived.

This study protocol presents the study design for each subgroup and describes the study population, the methodological approach (including data collection and data analysis), the relevant outcomes and the quality criteria of the study, and discusses the expected benefits and limitations. The aim is to give an overview of the different sub-studies (modules), to describe contexts and to present the methodological approach.

## Methods and analysis

### Design and setting of the study

The mixed-methods study will be conducted in multiple phases. Quantitative and qualitative methods will be performed in a parallel or in a sequential order (18). Standardized surveys are used to obtain a representative and comprehensive understanding of the different stakeholder groups and to make the answers comparable. The qualitative methods are suitable to contextualize and interpret the data appropriately (18). The methodological openness of qualitative methods also allows aspects to be included that arise from everyday conditions and are accordingly not yet theoretically accessible at the beginning of the study, for example organizational barriers in everyday practice (19). Methodological triangulation allows the results of quantitative and qualitative interviews to be validated against each other (20).

**FIGURE 2:**
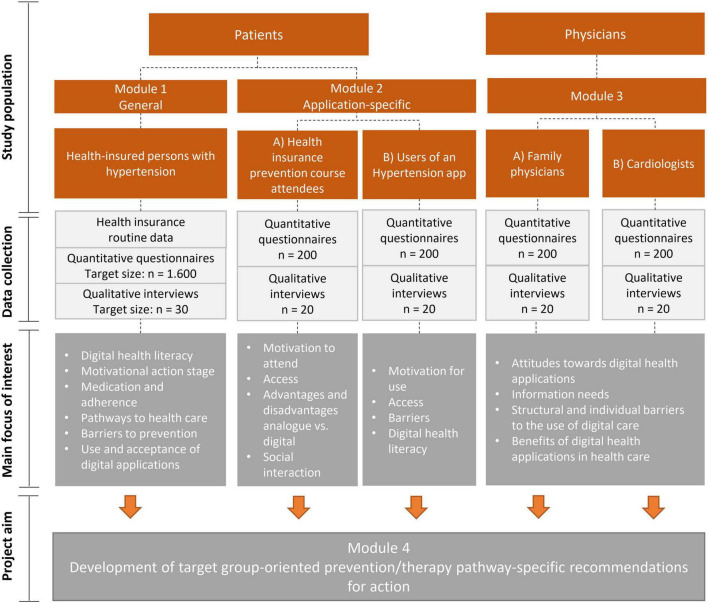
Study design.

Figure 2 shows a summary of the study design, methods and the main focus of interest in each module. Since the focus is on the user perspective, we will include patients with arterial hypertension, users of digital health services and physicians. The timeline of recruitment is presented in Table 1.

**TABLE 1:** Dates of recruitment.

Substudy	Dates of recruitment
Module 1 interviews	Q3 2022 – Q1 2023 (6 months)
Module 1 questionnaires	Q4 2023 – Q1 2024 (4 months)
Module 2a interviews and questionnaires	Q4 2022 – Q3 2023 (11 months)
Module 2b interviews and questionnaires	Q4 2022 – Q3 2023 (11 months)
Module 3a interviews	Q4 2022 – Q2 2023 (5 months)
Module 3a questionnaires	Q4 2023 – Q1 2024 (4 months)
Module 3b interviews	Q4 2022 – Q2 2023 (5 months)
Module 3b questionnaires	Q4 2023 – Q1 2024 (4 months)

Q, quarter. Q1 = Jan–Mar, Q2 = Apr–Jun, Q3 = Jul–Sep, Q4 = Oct–Dec.

The study will be conducted in Germany with a regional focus on the metropolitan area of Berlin and the rural, sparsely populated federal states of Brandenburg and Mecklenburg-Western Pomerania.

### Selection of subjects

The sub-study specific inclusion criteria are summarized in Table 2. Exclusion criteria are age under 18, lack of consent and considerable limitations to speak and understand German fluently.

**TABLE 2:** Additional inclusion criteria.

Substudy	Inclusion criteria
Module 1 interviews	Diagnosis of arterial hypertension
Module 1 questionnaires	Diagnosis of arterial hypertension and health-insured by the AOK Nordost
Module 2a	Participation in a behavioral prevention course offered by AOK Nordost
Module 2b	Use of a hypertension app within the last 2 years
Module 3a	Current work as a family physician
Module 3b	Current work as a specialist in cardiology

In module 1, insured persons of the statutory health insurance company AOK Nordost with arterial hypertension will be surveyed on preferences and barriers to the use of (digital) prevention measures (*n* = 1,600 questionnaires). Questionnaire data will be linked to routine data to analyze further health-related factors. Prior to the AOK Nordost survey, a preparatory qualitative interview study will be conducted with hypertension patients recruited through clinics and practices (*n* = 30).

In module 2a, users of (digital) prevention services offered by AOK Nordost will be surveyed on their motivation to participate in the interventions, the advantages and disadvantages of digital vs. analog measures, and possible barriers to the services (*n* = 200 questionnaires, *n* = 20 interviews).

In module 2b, users of the hypertension-specific app “Hypertonie.App” (21) will be interviewed about their motivation for using the app, about possible restraining factors when using a hypertension app and how they became aware of the app (*n* = 200 questionnaires, *n* = 20 interviews). The app includes features for documenting blood pressure, personalized health information, breathing exercises, and reminder functions.

In module 3, family physicians (3a) and cardiologists (3b) will be surveyed on their attitudes toward digital health applications, structural and individual barriers, and information needs in medical practice (*n* = 400 questionnaires, *n* = 40 interviews).

### Sample size calculation

A sample number of *n* = 200 is required per subgroup. The sample number is calculated according to an *a priori* power analysis (G*Power) for the χ^2^ test for dichotomous variables with the following assumptions: moderate effect (*w* = 0.2), α = 0.05, power (1-β) = 0.80, Df = 1 (required sample size *n* = 197).

In Module 1, eight subgroups will be identified by age, gender, and rural vs. urban residence (per subgroup *n* = 200, total *n* = 1,600).

### Outcomes

The DiPaH study will collect data through questionnaires and interviews on a one-time basis. The relevant outcomes are digital health literacy [eHEALS (22, 23)], health literacy [HLS-EU-Q6 (24, 25)], motivation action stage [HAPA Assessment (26, 27)], self-concept related to information and communication technology (28), perception of usability [System Usability Scale (29, 30)], guideline-adherent medication (4), adherence [MMAS-8D (31, 32)]. The outcomes and measurement tools are presented in Table 3. In addition, the following outcomes will be explored in all modules: attitudes toward digital prevention, use of digital prevention, motivation to use digital prevention, barriers to digital prevention, perceived advantages and disadvantages of digital prevention, information needs, and – just for patients – pathways to health care. For these outcomes, module-specific items will be developed and piloted by the research team. In addition, these themes will be explored in greater detail in the qualitative interviews.

**TABLE 3:** Outcomes and measurement tools.

Outcome	Measurement tools	References	Items	Reliability	Module				
					1	2a	2b	3a	3b
Digital health literacy	eHEALS	Norman and Skinner (22), German: Soellner et al. (23)	8	Cronbach’s α = 0.828 to Cronbach’s α = 0.877	x	x	x		
Health literacy	HLS-EU-Q6	Sørensen et al. (24), Short Version: Pelikan et al. (25)	6	Cronbach’s α = 0,803	x	x			
Motivation action stage	HAPA Assessment: stage assessment	Lippke et al. (27)	1	Sensitivity = 70% to 80% Specificity = 80% to 87%	x		x		
Self-concept related to information and communication technology	General ICT-SC	Schauffel et al. (28)	5	Cronbach’s α = 0.95	x	x			
Perception of usability	System Usability Scale	Brooke (30), Bangor et al. (29)	10	Cronbach’s α = 0.911			x		
Adherence	MMAS-8	Morisky et al. (32), German: Arnet et al. (31)	8	Cronbach’s α = 0.41	x				
Guideline-adherent medication	ESC guideline criteria	Williams et al. (4)			x				

Input variables in all modules will be sociodemographic characteristics of patients (age, gender, rural vs. urban residence, federal state, education, income) and physicians (age, gender, rural vs. urban location of medical practice, federal state, experience). In Modules 1 and 2b, health-related characteristics will be collected in more detail (current medication, disease status, comorbidities). In Module 2a, the interactivity of the course is additionally assessed as an input variable.

### Statistical analyses

First, the data from the standardized questionnaires will be presented descriptively (absolute and relative frequency, median and standard deviation). In further steps, correlation analyses will be performed. The correlation between two binary variables is analyzed with the χ2-test. For continuous outcome variables and binary influence factors, *t*-tests will be performed and means and 95% confidence intervals will be reported. Regression analyses will be performed to analyze possible associations between patient characteristics (such as sociodemographic variables) and outcomes of interest (e.g., digital health literacy).

### Qualitative analyses

For the qualitative part of the study, problem-centered interviews will be conducted. In this type of exploratory interview, the theoretical concepts are changeable in the research process. Thus, the method enables to integrate the subjective perspectives, experiences and contextual conditions of the participants. In addition to the interview guide, short questionnaires will be applied to collect sociodemographic and health-related characteristics.

The interviews will be recorded and transcribed. The qualitative data from the interviews and from the free text answers in the questionnaire will be analyzed with the qualitative content analysis according to Kuckartz (33). This allows a rule-driven reduction and systematization of the data. The aim is to develop a comprehensive category system. For validation of the category system, a member check will be performed (34).

### Data capture and storage

In modules 1 and 2a, the insured persons receive the questionnaire from their health insurer AOK Nordost with a stamped return envelope. By giving written consent, participants in module 1 agree to the linking of questionnaire data with the billing data of the health insurance funds. The pseudonymization of the data will be carried out by a trust center on the basis of a coding list. The trust center is separated from the evaluating unit in terms of space, technology, and staff. In modules 2 and 3 online participation will also be possible. For this, participants will receive a link to the questionnaire on the online platform “unipark” (35).

To receive the incentive (money or gift card), participants need to consent that their contact details will be used for this purpose. Written consent is also required for the audio recording of the interviews. The interviews will be coded with a pseudonym before the analysis. Contact information and consent will be kept separate from research data.

Research data will not contain personal data that would allow the identification of individuals. The data will be stored securely on the server of the Brandenburg Medical School or on local computers.

### Data quality

To ensure high data quality, several data validation checks will be performed: checking for correct data type, numeric value ranges, data format, missing data, and consistency of the chronology in the dates. The completeness of the data is checked per case. The quality of the interview data will be controlled by a member check (36).

## Discussion

Global health systems today confront fundamental challenges in providing optimal care due to aging populations, shortages of health workers, financing, accessibility and affordability of cardiovascular disease medicines and services. Digital health technologies have the potential to overcome these challenges (37). Consequently, the establishment of e-health to improve widespread cardiovascular care is a central goal of the ESC. In terms of arterial hypertension, a digital hypertension program and m-health tools have already been shown to lead to significant improvement in blood pressure control rates and lifestyle change compared to standard care (38, 39). However, before successful and widespread implementation, there is still a great need for research on hindering and supportive elements in special patient populations (11). To our knowledge, DiPaH is the largest health services research study on the topic of digital cardiovascular prevention measures and will fill this important knowledge gap. Different patient cohorts will be characterized according to their prevention needs and possible barriers to the use of digital prevention measures. At the same time, physicians will be involved as a main contact for their patients in order to identify structural and individual obstacles as well as information needs in medical practice. Based on this, practical recommendations will eventually be developed at the levels of patients, physicians, service providers, and health insurers to increase the implementation, accessibility, use of, as well as the adherence to digital prevention measures. These will enable hypertensive patients to take a more active role in their own care and will have the potential to significantly improve current clinical care pathways (11). We are also convinced that these findings will help to implement digital health-based care models for cardiovascular disease in general, beyond the application field of arterial hypertension.

### Strengths and limitations

The strengths of DiPaH include an integrative approach of a mixed-methods study protocol that comprehensively considers the perspectives of patients, physicians and participants of analog and digital prevention measures. Special attention is paid to rural and sparsely populated areas, age-specific effects and the influence of digital health literacy. This allows concrete recommendations to be derived for wider adoption and sustainable use of digital prevention interventions in cardiovascular medicine. A limitation of the study is that it is only conducted in Germany. But by using internationally validated questionnaires, relevant findings can also be obtained for other countries.

Even though we are only survey the users of a single hypertension app, the aim is not to evaluate this one app. Rather, in DiPaH we want to characterize the app users and find out why they use a digital hypertension app. Accordingly, a gain in knowledge is expected for other apps. In particular, it is assumed that barriers, motivations, attitudes, and pathways to health care are not app-specific.

However, the AOK Nordost is the largest statutory health insurance fund in north-eastern Germany with about 1.7 million insured and covers about 18% of the population in north-eastern Germany (approx. 7.82 million inhabitants, 2021 (40)). As there is no standardized electronic patient record in Germany so far, we will not use primary data from hospitals and health care facilities. Surveying participants *via* a health insurer has the advantage that there are no gatekeepers, e.g., physicians enrolling patients in the study and thereby selecting them (intentionally or unintentionally), and that the billing data is complete. The limitation of billing data (e.g., shows only picking up medications, not actually taking them) is compensated for by the use of questionnaires. The secondary data from the health insurance funds are available 9 months later. This time period was taken into account in the study design.

## Conclusion

The roadmap for digital health adoption in cardiology is challenging but imperative to address future challenges in cardiovascular medicine. DiPaH will make an important contribution by identifying the specific barriers to the adoption of digital health technologies for arterial hypertension and providing recommendations for overcoming them.

## Ethics statement

The study complies with good clinical practice in accordance with the Declaration of Helsinki and the laws and regulations applicable in Germany. The local Ethics Committee of the Brandenburg Medical School approved the study (Record number E-02-20220620). Due to the sequential approach of the mixed-methods design, the questionnaires in modules 1 and 3 will be modified based on the qualitative analysis. Therefore, an amendment will be requested from the Ethics Committee before the survey starts. If necessary, the relevant information in the German Clinical Trials Register will be updated.

## Author contributions

DB, SS, ML, MH, FM, SM, E-LB, EW, GF, OJ, and MM contributed to the study conception and design. SS, DB, and ML contributed to the obtaining funding. DB, SS, SM, FM, EW, JA, E-LB, KR, and OJ contributed to the collection of data. DB, SM, FM, EW, and E-LB contributed to the analysis of data. SS, DB, ML, MH, JA, and MM contributed to the supervision. DB and SS drafted and revised the manuscript. All authors read and approved the final manuscript.
